# A plant factory for moth pheromone production

**DOI:** 10.1038/ncomms4353

**Published:** 2014-02-25

**Authors:** Bao-Jian Ding, Per Hofvander, Hong-Lei Wang, Timothy P. Durrett, Sten Stymne, Christer Löfstedt

**Affiliations:** 1 Department of Biology, Lund University, Sölvegatan 37, SE-22362 Lund, Sweden; 2 Department of Plant Breeding and Biotechnology, Swedish University of Agricultural Sciences, SE-23053 Alnarp, Sweden; 3 Department of Biochemistry and Molecular Biophysics, Kansas State University, Manhattan, Kansas 66506, USA

## Abstract

Moths depend on pheromone communication for mate finding and synthetic pheromones are used for monitoring or disruption of pheromone communication in pest insects. Here we produce moth sex pheromone, using *Nicotiana benthamiana* as a plant factory, by transient expression of up to four genes coding for consecutive biosynthetic steps. We specifically produce multicomponent sex pheromones for two species. The fatty alcohol fractions from the genetically modified plants are acetylated to mimic the respective sex pheromones of the small ermine moths *Yponomeuta evonymella* and *Y. padella*. These mixtures are very efficient and specific for trapping of male moths, matching the activity of conventionally produced pheromones. Our long-term vision is to design tailor-made production of any moth pheromone component in genetically modified plants. Such semisynthetic preparation of sex pheromones is a novel and cost-effective way of producing moderate to large quantities of pheromones with high purity and a minimum of hazardous waste.

## Abstract (web summary)

Pheromones can be used as an environmentally friendly alternative to traditional pesticides. Here, the authors produce moth sex pheromones in *Nicotiana benthamiana* by transient expression and demonstrate that these pheromones are able to trap male moths as efficiently as their synthetic counterparts.

## Graphical abstract

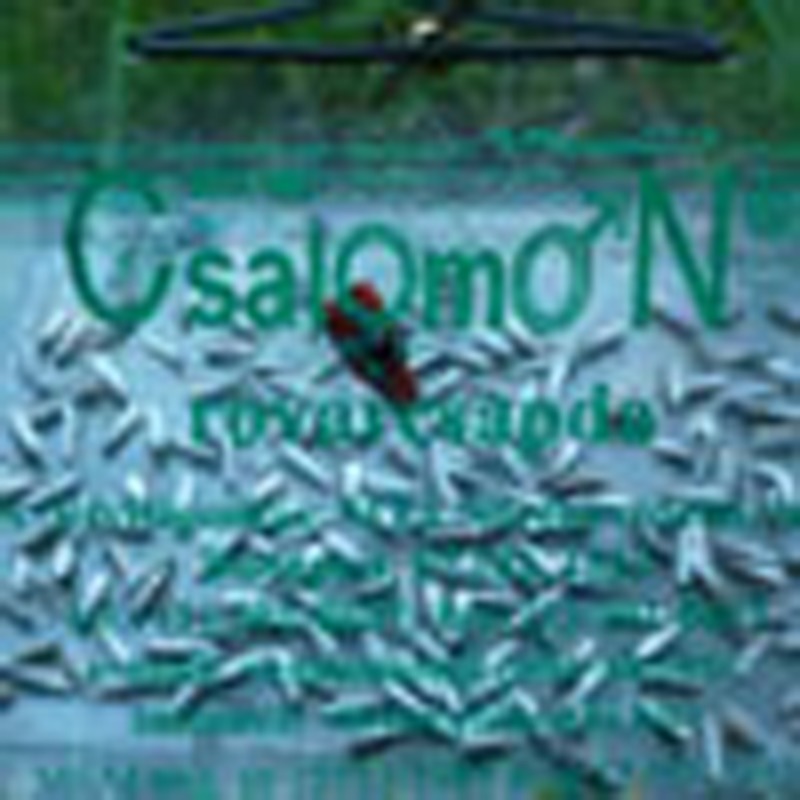

Pheromones are environmentally friendly alternatives to traditional pesticides for the control of insect pests and indeed synthetic pheromones are produced in large amounts for this purpose^1^. Current standard approaches to pheromone synthesis either require the use of hazardous chemicals or may result in the production of hazardous waste byproducts^2^,^3^. We propose to overcome the problems inherent to synthetic pheromone production by designing and developing an innovative green chemistry alternative while minimizing hazards^4^. Our strategy involves the use of a cost-effective plant factory expressing a suite of biosynthetic enzymes for production of moth pheromones.

Female moths emit species-specific pheromone component blends that attract males of the same species over long distances^1^. A majority of the identified moth pheromone compounds consist of fatty acid derivatives produced *de novo* in the pheromone gland^5^,^6^. Their biosynthesis typically involves desaturation of fatty acids, reduction to primary alcohols and further modification to produce acetates or aldehydes. Great advances have been made during the last 15 years with respect to our understanding of the molecular basis of moth pheromone biosynthesis^7^,^8^. Heterologous expression systems have allowed the confirmation of the function of many desaturases^9^,^10^,^11^,^12^,^13^ and fatty-acyl reductases (FARs)^14^,^15^,^16^,^17^. When expressed in yeast, the desaturases produce fatty acids of different chain lengths with double bonds in different positions. The functionally characterized FARs convert fatty-acyl moieties into fatty alcohols.

Transgenic plants have proven useful to express enzymes from insects. For example, transgenic plants produced a pheromone precursor upon introduction of a moth desaturase^18^ and an aphid alarm pheromone has been produced from endogenous plant sesquiterpene by expression of an (*E*)-β-farnesene synthase cDNA^19^. We wanted to take this approach further and exploit the molecular toolbox currently available to produce specific multicomponent moth pheromones in *Nicotiana benthamiana*. This requires the coordinated expression of several genes to be successful. Producing stable lines of transformed plants that assemble these multistep recombinant pathways remains a major challenge. An alternative is to express the genes in leaves transiently via infiltration of the abaxial air spaces with *Agrobacterium tumefaciens* cultures harbouring each expression construct plus viral silencing suppressor protein P19 that inhibits the host cells’ transgene-silencing apparatus^20^. This leaf-based expression format has been used in transient expression of the multicomponent metabolic pathway of polyunsaturated fatty acid metabolism^21^.

Here we first produce (*Z*)-11-hexadecenol (Z11-16:OH), (*E*)-11-tetradecenol (E11-14:OH), (*Z*)-11-tetradecenol (Z11-14:OH) and their corresponding acetates, by introducing all the necessary and sufficient genes into *N. benthamiana* plants. These acetates and alcohols have been identified as pheromone components in hundreds of moth species^22^. Specifically, we then produce plant-derived mimics of the sex pheromones of two species of small ermine moths, *Yponomeuta evonymella* and *Y. padella*^23^ and compare their behavioural activity to conventionally produced pheromones. Our series of proof-of-principle experiments demonstrate the feasibility of production of behaviourally active moth pheromones in plants.

## Results

### Assembly of moth pheromone biosynthetic pathways

**Figure 1:**
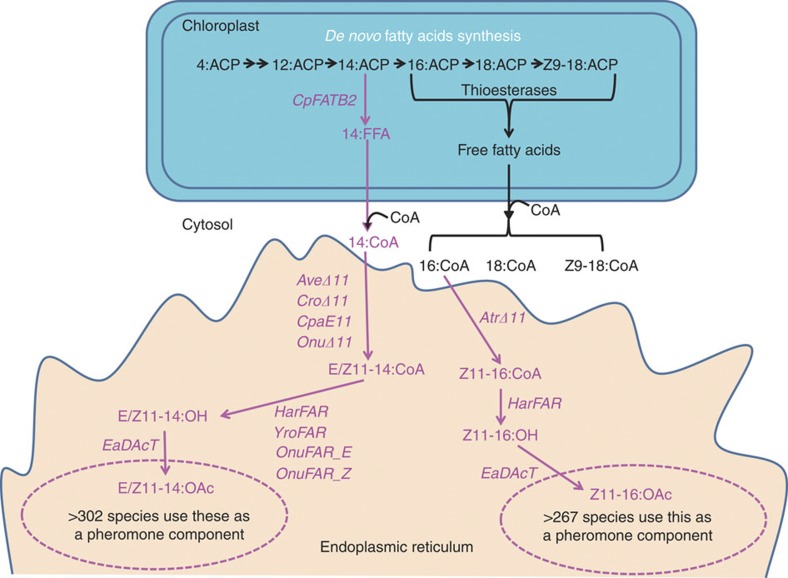
Production of moth pheromones in *N. benthamiana* leaf cells by transient expression of introduced genes. Introduced enzymes and pathways are depicted in magenta. 16C pheromones are produced from preexisting cytosolic pool of 16:CoA whereas 14C pheromones are produced from a cytosolic 14:CoA pool created by introduction of a *Cuphea palustris* thioesterase intersecting chloroplast *de novo* acyl synthesis at 14C level. The genes for the enzymes involved in the pheromone production in this study were cloned from various moth and plant species: CpFATB2, *Cuphea palustris* 14:ACP thioesterase; AveΔ11, *Argyrotaenia velutinana* desaturase; CroΔ11, *Choristoneura rosaceana* desaturase; CpaE11, *Choristoneura parallela* desaturase; OnuΔ11, *Ostrinia nubilalis* desaturase; AtrΔ11, *Amyelois transitella* desaturase, HarFAR, *Helicoverpa armigera* acyl reductase; YroFAR, *Yponomeuta rorellus* acyl reductase, OnuFAR_E/Z, *Ostrinia nubilalis* acyl reductase, EaDAcT, *Euonymus alatus* acetyltransferase. Acyl intermediates in the pathway (also throughout the article) are given as short forms, for instance, E/Z11-14:CoA refers to the fatty-acyl coenzyme A with a chain length of 14-carbon atoms and a double bond at Δ11 position in ‘E’ or ‘Z’ configuration. ACP, acyl carrier protein; FFA, free fatty acid; OH, fatty alcohol; OAc, acetate.

In plant leaf cells, *de novo* fatty acid biosynthesis takes place in the chloroplasts. The sequential, two carbon atom elongation of the acyl chain esterified to acyl carrier protein (ACP) is catalysed by the fatty acid synthase complex. The majority of the acyl groups are cleaved from ACP at 16 and 18 carbon lengths by plastidial thioesterases, transported through the plastidial envelope and acylated to CoA at the outer envelope. The acyl-CoAs are then utilized for the synthesis of extraplastidic lipids by enzymes localized in the endoplasmic reticulum (ER)^24^. The enzymes of pheromone biosynthesis used in this study also reside in the ER in their natural environment^25^. Taking this into consideration, we thus took the following approach to pheromone production in *N. benthamiana* leaves (Fig. 1): By introducing a plastidial thioesterase, CpFATB2, derived from *Cuphea palustris,* that terminates the fatty acid biosynthesis by mainly hydrolyzing the thioester bond of myristoyl-ACP (14:ACP)^26^, we increased the amount of myristic acid exported from the plastid, thereby creating a cytosolic pool of 14:CoA available for pheromone biosynthesis. We also introduced Δ11 desaturases that specifically recognizes 14:CoA and 16:CoA to produce (*E/Z*)-11-tetradecenoyl-CoA (E/Z11-14:CoA) and (*Z*)-11-hexadecenoyl-CoA (Z11-16:CoA). FARs from several moth species could then be introduced to reduce the E/Z11-14:CoA and Z11-16:CoA into E/Z11-14:OH and Z11-16:OH. Finally, a plant-derived acetyltransferase gene (*EaDAcT*)^27^ was expressed to transfer an acetate group to the primary alcohols and form the (*E/Z*)-11-tetradecenyl acetates (E/Z11-14:OAc) and (*Z*)-11-hexadecenyl acetate (Z11-16:OAc).

To evaluate the feasibility of our approach, we first expressed candidate genes individually and in combinations for each of the biosynthetic steps required for pheromone production to take place in the leaves. In this way, 11 genes were characterized (Table 1). Fifty gene combinations were evaluated out of which 34 yielded products of interest. The amount of myristic acid (14:acyl) substrate available to insect pheromone component biosynthesis in *N. benthamiana* is very low. Upon expression of the thioesterase CpFATB2, the plants generally produced more than 200 μg of myristic acid per gram fresh leaf tissue, whereas the amount in control plants was less than 2 μg.

Next, we tested five desaturases exhibiting various specificity and functionality in our leaf chassis. It was shown previously that insect-derived desaturases involved in pheromone biosynthesis can be successfully expressed in tobacco^18^. The OnuΔ11 (ref. 12) showed no detectable activity in our leaf expression system, whereas the other four conferred on the plant the ability to produce amounts of unsaturated products ranging from 30 to 600 μg per gram fresh leaf tissue. Relative to the respective saturated precursor, the amount of product found was in the range of 9–50%. Among these four desaturases, we selected the AtrΔ11 and AveΔ11 (ref. 9) as candidates for subsequent larger scale production of pheromone. AtrΔ11 showed high substrate specificity towards 16:CoA and AveΔ11 specifically interacted with 14:CoA to produce a mixture of E11-14:CoA and Z11-14:CoA. The activity of CroΔ11 (ref. 10) was very similar to that of AveΔ11 but CroΔ11 produced a somewhat higher ratio of E11- to Z11-14:CoA, which was not suitable for the production of small ermine moth pheromones.

In the third step, we evaluated four FARs that have previously been shown to be active in moth pheromone biosynthesis^15^,^16^,^17^. The OnuFAR Z^15^ and E^15^ resulted in very low product yield when expressed in plants. HarFAR^17^ showed higher conversion rates than YroFAR^16^. The reduction of fatty-acyl precursors to the corresponding fatty alcohols was much less efficient compared with the desaturation. Efforts were made to optimize the conversion rates by engineering variants of FARs. Of all the tested modifications (Methods), only the ER retention signal (*KKYR*), attached to the C-terminal end of the enzyme, improved the alcohol production over the original version (Table 1). Therefore, *HarFAR_KKYR* was selected as the most suitable candidate for the larger scale pheromone production experiment to be performed.

**Figure 2:**
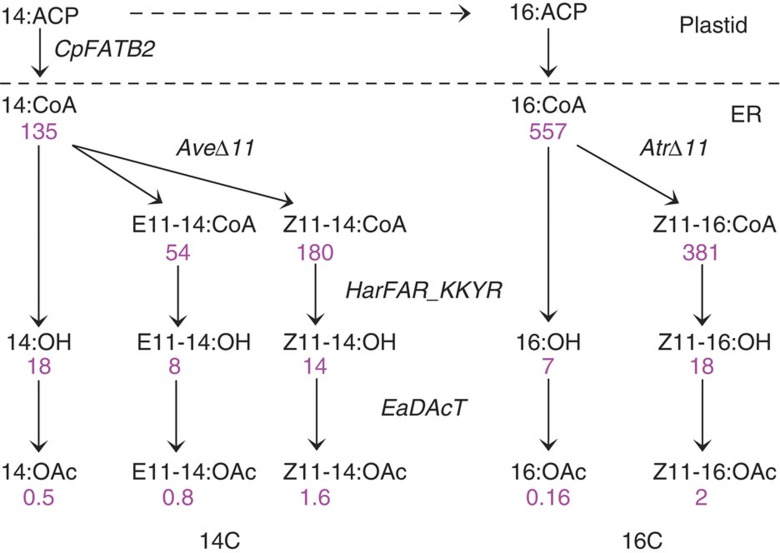
Optimized pathways for moth pheromone production. Expression of four (*CpFATB2, Ave*Δ*11, HarFAR, EaDAcT*) or three (*Atr*Δ*11, HarFAR, EaDAcT*) genes in two different tobacco plants, led to the production of 14-carbon (14C) or 16-carbon (16C) pheromone components. Amounts (numbers in magenta) are μg of products recovered from one gram of fresh leaf tissue in the respective treatments.

**Figure 3:**
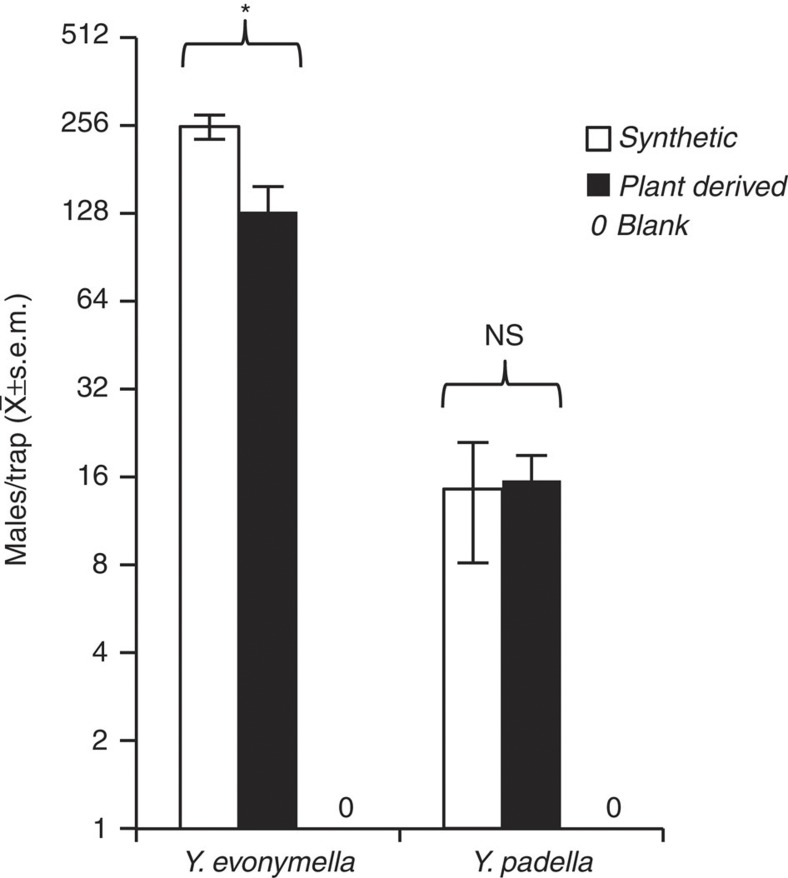
Test of attractiveness of plant-derived moth pheromones. Trap catches of males of two small ermine moth species obtained with synthetic pheromone (positive control), plant-derived pheromone and blank traps (negative control) (*N*=5). Plant-derived pheromones were prepared by acetylation of corresponding alcohols produced from the 14C and 16C treatment, respectively. The treatment producing 14C compounds was used alone for trapping *Yponomeuta evonymella*, and a mixture of the two treatments producing 14C and 16C compounds was used for trapping *Y. padella*. The synthetic baits for *Y. evonymella* contained 14:OAc/E11-14:OAc/Z11-14:OAc in a blend ratio of 187:45:100, and the synthetic baits for *Y. padella* contained 14:OAc/E11-14:OAc/Z11-14:OAc/Z11-16:OAc in a ratio of 187:45:100:400. The baits of plant-derived compounds contained above-mentioned acetates and minor by-products. Moth catches were species specific with both synthetic and plant-derived pheromone. In the case of *Y. evonymella*, the plant-derived pheromone was not as effective as the synthetic pheromones (*t*-test, *P*=0.012), whereas for trapping of *Y. padella*, the plant-derived pheromones showed no difference from the synthetic pheromones (*t*-test, *P*=0.896). The blank control traps did not catch any moths. Error bars represent s.e.m. (standard error of mean). NS, not significant; *, significant (*P*<0.05).

In the fourth and final step, a plant-derived diacylglycerol acetyltransferase (EaDAcT), cloned from the Burning Bush (*Euonymus alatus*)^27^ was tested, as no acetyltransferase involved in moth pheromone biosynthesis has been characterized so far. EaDAcT is similar to the Jojoba wax synthase, which is a fatty-acyl-CoA: fatty alcohol acyltransferase, and it may therefore possess the ability to acetylate long-chain alcohols. When expressed in our leaf transient expression system, it resulted in 5–10% conversion rate of 14C and 16C alcohol substrates (Table 1, Fig. 2). We considered the acetate production to be on the low side, both in terms of absolute amounts and in relation to the amount of precursor alcohol and for extraction and implementation in the trapping of moths (Fig. 3). This particular step will eventually require further improvements and will most likely benefit from the future characterization of an insect enzyme.

### Attractiveness of plant-derived moth pheromones

Using the most promising candidate genes, we aimed at specifically producing the *Y. evonymella* and *Y. padella* pheromones in amounts sufficient for attraction of male moths in the field. *Y. evonymella* and *Y. padella* are attracted to Z11-14:OAc/E11-14:OAc/14:OAc=100/20/60 and Z11-14:OAc/E11-14:OAc/Z11-16:OAc=100/34/400, respectively. Although they lack minor components necessary for maximal attraction, these three-component mixture blends of synthetic compounds have still been confirmed to be highly attractive and species specific when tested in field experiments^23^,^28^. To obtain enough pheromone for trapping of moths in the field, all the leaves (*ca*. 10) on a *N. benthamiana* plant were infiltrated. One plant was infiltrated with *P19*, *CpFATB2*, *Ave*Δ*11* and *HarFAR* to produce a mixture of 14C alcohols and another plant was infiltrated with *P19*, *Atr*Δ*11* and *HarFAR* to produce 16C alcohols. The alcohol fractions from the leaves were isolated and acetylated chemically. The 14C alcohol-producing plant yielded more than 500 μg of 14C acetates plus a smaller amount of saturated 16:OAc; the 16C plant produced 1,280 μg Z11-16:OAc and a smaller amount of 16:OAc as determined by GC–MS analysis (Table 2).

For trapping of *Y. evonymella*, the acetate mixture from the 14C alcohol-producing plant was used as it was, whereas a 1:4 mixture of the acetates from both 14C and 16C alcohol-producing plants were used for trapping of *Y. padella*. The behavioural activity of the plant-derived acetate mixtures was compared with the activity of conventionally produced synthetic three-component pheromone lures (positive controls) and blank traps (negative controls). Catches of *Y. evonymella* were very high. Traps baited with plant-derived pheromone attracted on average 130 males per trap, approximately half the catches obtained with conventional produced pheromone components. In the case of *Y. padella,* the population was obviously not as abundant in the trapping area but for this species the plant-derived pheromone was not different from the conventionally produced pheromone in attractiveness. Blank traps trapped no males of either species (Fig. 3).

## Discussion

Attraction of male moths to traps baited with plant-derived pheromones was high and specific, even if the 45/100 ratio of E11-14:OAc/Z11-14:OAc produced in our treatments is not optimal for attraction of *Y. padella* and *Y. evonymella*. Based on previous studies, better attraction for both species would be obtained with a lower proportion of the E isomer^23^. However, we predict that in the near future, it will be possible to modify the ratio between plant-produced isomers by engineering the desaturase and reductase genes. We have already demonstrated that substitution of one amino acid can cause remarkable changes in specificity of a moth FAR^8^. The genes and molecular techniques available to us thus constitute a powerful toolbox from which we can choose the elements to be used in the construction of genetically modified plants producing optimal pheromone component ratios.

Modifying and optimizing the acetate/alcohol ratio is more challenging. Most moth pheromones contain acetates as major components. Too high amounts of corresponding alcohols may significantly reduce or even completely abolish attraction. This means that either the conversion of alcohols into acetates has to be almost complete or we have to be able to control the relative release of alcohols and acetates from the plants. For the time being, we do not know anything about the release of moth sex pheromone components from intact plants. Even though transient expression has been shown to be an efficient system for generating various products^29^, stable transformation would be required to eliminate applications of expression constructs and to obtain a system where a larger part of the biomass produces the product of interest over a longer time. Optimization of expression with regards to promoter strength and temporal control or induction would be of importance in the optimization of product yield. Endogenous temporal control would require identification of a promoter that has an onset at a desired time point during plant development. In the case of inducible expression, several systems exist which involve application of low-cost compounds to induce gene expression, one example of such an inducer being methoxyfenozide^30^. Modifications of plant endogenous lipid metabolic processes to build an efficient flow from saturated substrates via desaturation, reduction and down-stream modifications such as acetylation, will involve coordinated expression of multiple constructs^31^ in multiple copies. This is a research challenge, requiring the production of numerous transgenic lines and selection of elite events.

The current study paves the way for future production of moth pheromones in plant factories. Our experiments convincingly demonstrate that it is possible to produce highly attractive and species-specific moth pheromones making use of genetically modified plants (Fig. 2). This approach is just in its infancy, but the results are already promising. We now aim at developing tools to optimize ratios of the plant-produced pheromone compounds, increasing the yield and controlling what is released from the plants. Our long-term vision is to design tailor-made production of any moth pheromone component in stably transformed plants. Such sustainable preparation of sex pheromones from plants will be a novel and cost-effective way of producing moderate to large quantities of pheromones with high purity and a minimum of hazardous waste. Moving from transient expression to stable transformants will further improve the system. Genetically modified plants may eventually be used in intercropping as natural dispensers of insect pheromones and as part of a push-pull strategy^32^,^33^, provided that they can be shown to have no negative environmental side effects. Our proposed strategy is innovative and environmentally friendly. Although it contains fundamental research challenges, it has the potential to become an economically sound part of many integrated pest management programs.

## Methods

### Plasmid constructs for transient expression

All the genes, including: *CpFATB2*, *Atr*Δ*11*,*Onu*Δ*11*, *YroFAR*, *HarFAR*, *OnuFAR_E/Z* and *EaDAcT* were amplified from cDNA with primers spanning from the start codon to the stop codon of the ORF, except *Ave*Δ*11*, *Cro*Δ*11* and *CpaE11*, which were synthesized and codon optimized for the *N. benthamiana* codon bias (Invitrogen, Life Technologies). Truncation, gene fusion, ER targeting signal and retention signal attachments were achieved by PCR and fusion PCR^34^, as presented in the Methods.

All genes and their modified versions were cloned into plant expression vector pXZP393 by Gateway recombination cloning technology (Invitrogen). After confirming the integrity of the constructs by sequencing, the expression clones were introduced into *Agrobacterium tumefaciens* strain GV3101 (MP90RK) by electroporation (1500 V mm^−1^, 5 ms, Eppendorf 2510).

In order to inhibit the host cells’ transgene-silencing apparatus and extend transgene expression over a longer period of time with a higher degree of expression, the viral silencing suppressor protein P19 was co-introduced into the *Agrobacterium* mixtures^21^.

### Outline of all the molecular manipulations performed on FARs

The used insect FARs display amino-acid sequence in the last ~100 aa of the C-terminal region, which could form a transmembrane region or membrane anchor as predicted using TMHMM^35^. As a first modification (A), this region was truncated as it had been shown that a similar truncation of mouse FAR1, MmFAR1Δc, had rendered the heterologous expressed enzyme highly active (HarFAR was truncated at 352L-V353; YroFAR was truncated at 353K-F354)^36^. In a second modification (B), the C-terminal region was replaced by a corresponding region of either MmFAR1Δc or a *Marinobacter aquaeolei* VT8 FAR^37^ (HarMmuFAR: SACNP—FHWGE; HarMaqFAR: ACNPI—SLGEF; YroMmuFAR: GTTNP—FHWGE; YroMaqFAR: GTTNP—ISLGE; aa sequence at junction are shown). In a third set of modifications (C), an ER retention signal was added to the C-terminal (HarFAR_KKSY: KKSYE->KKSY; HarFAR_KKYR: KKSYE->KKYR; YroFAR_KKYR: attaching KKYR to the C-terminal).

Although there is no direct evidence showing that the insect-derived fatty acyl desaturases involved in sex pheromone biosynthesis are present in the ER, their four transmembrane regions strongly suggest they are located in the ER^38^. With the aim of bringing the FARs nearer to the desaturases, different versions of gene fusions were produced: (Atr1-267)_HarFAR_KKYR: HRLWAH—MVVLTS (this construct fuses the first 89 aa of AtrΔ11 to HarFAR_KKYR, that contains the ER targeting signal and first transmembrane domain); AtrΔ11_(Atr1-267)_HarKKYR (this construct fuses AtrΔ11 with HarFAR_KKYR, with a long spacer, that is the first 89aa from AtrΔ11); AveYroFAR (this construct fuses AveΔ11 with YroFAR with no spacer); AtrHarFAR (this construct fuses AtrΔ11 with HarFAR with no spacer).

### *Agrobacterium* infiltrations of *Nicotiana benthamiana*

Ten ml of *Agrobacterium* solution containing individual gene constructs were grown at 28 °C with LB medium supplemented with appropriate antibiotics overnight in a 300 r.p.m. (revolutions per minute) incubator. Acetosyringone (final conc. 100 μM) was added to the overnight culture and grown for an additional 2–3 h to induce virulence genes encoded by the *Agrobacterium* genome. Bacteria were spun down at 3,000 *g* for 5 min at room temperature and resuspended in infiltration buffer (5 mM MgCl_2_, 5 mM 4-morpholineethanesulphonic acid, 100 μM acetosyringone, pH 5.7). Optical density (*A*_600 nm_) of each *Agrobacterium* culture was measured and the final concentration of each culture was adjusted equally to *A*_600 nm_=0.2, in a total volume of 20 ml, by infiltration buffer as described before. The final mixture of *Agrobacterium* cells was drawn up into a 1 ml syringe (without needle) and infiltrate into the underside of a suitable leaf, with a gentle squeeze on the plunger and modest pressure on the leaf using a finger, the *Agrobacterium* solution was forced into the mesophyll spaces wetting the leaf. Three entire leaves of similar age, from 1-month-old *N. benthamiana* plants were infiltrated. For production of fatty alcohols for pheromone traps, all the leaves from one *N. benthamiana* plant were infiltrated. The plant was watered generously and maintained in growth chamber for 4 days.

### Lipid analysis

Total lipids were extracted from *ca*. 1 g of leaf tissue using 3.75 ml of methanol/chloroform (2:1, v/v), in a glass tissue grinder. The crude extract was transferred to a glass tube and the grinder was rinsed with 1.25 ml of chloroform, which was then added to the extract. One ml of acetic acid (0.15 M) and 1.25 ml of water were added to the tube producing a biphasic mixture. Tubes were vortexed vigorously and centrifuged at 550 *g* for 2 min. The *ca*. 2.5 ml of chloroform phase containing the total lipids was transferred to a new glass tube.

Fatty acid methyl esters (FAMEs) were prepared from a portion of this total lipid extract. Thus, 100 μl of the extract was transferred to a new glass tube, and 10 μl (1 nmol μl^−1^) internal standard 17:Me was added. The solvent was evaporated under a gentle nitrogen flow. Then 1 ml of sulphuric acid (2% in methanol, v/v) was added, and the reactant was vortexed vigorously followed by 1-h incubation at 90 °C. After incubation, 1 ml of water was added, the mixture was vortexed and then 1 ml of hexane was added to extract the FAMEs.

Fatty alcohols (OH) and acetates (OAc) from the total lipid extract were separated by thin-layer chromatography (TLC). Ten μl of 17:OH (1 nmol μl^−1^) and 3 μg of Z8-13:OAc were first added as internal standards to 300 μl of total lipid extract, which was then concentrated to *ca*. 50 μl under gentle nitrogen flow. The concentrated sample was subsequently loaded onto a TLC plate (Silica gel 60, Merck, Germany) and separated with a mobile phase of hexane/diethyl ether/acetic acid (85:15:1, v/v/v). The bands were visualized by spraying water, and target gel areas were collected separately into 4-ml vials and extracted with 3.75 ml of methanol/chloroform (2:1, v/v) in sonication bath for 15 min. The extract was then centrifuged at 1,685 *g* for 3 min. The supernatant was transferred to a new tube and 1 ml acetic acid (0.15 M), 1.25 ml of chloroform and 1.25 ml of water were added to partition the lipids into chloroform. The chloroform phase containing alcohols or acetates, were transferred into new tubes and evaporated to dryness, followed by adding 40 μl of hexane to dissolve the compounds.

FAMEs, alcohols and acetates were analysed and quantified by GC–MS on a Hewlett-Packard 6890GC coupled to a mass detector HP 5975, operated in electron impact mode (70 eV). All compounds were identified by comparison of their mass spectra and retention times to those of corresponding synthetic standards available from our laboratory collection. The GC was equipped with a polar HP-INNOWax capillary column (100% polyethylene glycol; 30 cm*0.25 mm*0.25 μm, Agilent Technologies), and helium was used as carrier gas (average velocity: 33 cm s^−1^). The injection port was configured in splitless mode and the oven temperature was set to 80 °C for 1 min, then increased at a rate of 10 °C min^−1^ up to 210 °C, followed by a hold at 210 °C for 15 min, and then increased at a rate of 10 °C min^−1^ up to 230 °C followed by a hold at 230 °C for 20 min; the transfer line temperature was 280 °C. Data were analysed by the ChemStation software (Agilent, Technologies, USA).

### Field trapping experiment

All the leaves (about 20 g of fresh weight) on one *N. benthamiana* plant, in two combinations named plant #72 and plant #76 were infiltrated to prepare sufficient amounts of plant-derived pheromone for field trapping. For plant #72, which produced 14:OH, Z11-14:OH, E11-14:OH and 16:OH, the gene constructs were *P19, CpFATB2, Ave*Δ*11* and *HarFAR*. For plant #76, which produced 16:OH and Z11-16:OH, the gene constructs were *P19, Atr*Δ*11* and *HarFAR*. The fatty alcohols in the chloroform/methanol extracts of the leaves were isolated by TLC fractionation, and subsequently extracted by methanol/chloroform from the recovered silica gel. The alcohol extract was evaporated to dryness under gentle nitrogen flow, and the residues were used for preparing corresponding acetates by adding 1 ml of acetyl chloride and incubating at room temperature for 20 min after a brief vortex mixing. After acetylation, the solvent was evaporated under gentle nitrogen stream and 1 ml of hexane was added to dissolve the acetylated products, which were subsequently washed by 5% NaHCO_3_ and water, and finally dried over anhydrous Na_2_SO_4_.

The attractiveness of *N. benthamiana-*derived pheromones to male ermine moths was investigated in southern Sweden, in the vicinity of Lund on pastureland with shrubs of *Crataegus sp., Euonymus europeus* and *Prunus spinosa* (*Y. padella* 10^th^–24^th^, Aug, 2012), or along paths in a park with rich abundance of *P. padus* trees (*Y. evonymella*, 3^rd^–19^th^, Aug, 2012). Transparent plastic delta traps with sticky inserts (Csalomon, Budapest, Hungary) were used for the trapping experiments (five replicates for each treatment). Prior to loading the plant-derived pheromone compounds on the red rubber septa (Wheaton Science Products, Millville, NJ, USA), the absolute amounts and blend ratios were checked by GC–MS. A dose of 100 μg total acetates per septum was applied. Traps baited with synthetic acetates in same amounts and blend ratios were used as a positive control. The daily catch numbers were recorded and traps were redistributed within blocks after each check.

### Statistical analysis

Data were processed by Excel 2011 and Minitab13. Before conducting the test, normality tests of the data were performed. Two sample *t*-tests were used, to compare the moth catches of plant-derived pheromones and synthetic pheromones, the fatty alcohol productions from wild-type HarFAR and modified HarFAR.

## Author contributions

B.-J.D., P.H., S.S., and C.L. designed research; B.-J.D., H.-L.W., and C.L. performed research; P.H. and T.P.D. contributed new reagents/analytic tools; B.-J.D., P.H., S.S., and C.L. analysed data; B.-J.D. and C.L. wrote the paper. All authors read and improved the final version of the manuscript.

## Additional information

**Accession codes:** Sequence data for the following genes have been deposited in the GenBank/EMBL/DDBJ nucleotide core database under the accession codes CPU38189 (for *CpFATB2*), AF416738 (for *Ave*Δ*11*), AF545481 (for *Cro*Δ*11*), AF518014 (for *CpaE11*), AF441221 (for *Onu*Δ*11*), JX964774 (for *Atr*Δ*11*), JF709978 (for *HarFAR*), GQ907234 (for *YroFAR*), FJ807735/FJ807736 (for *OnuFAR_E/Z*), GU594061 (for *EaDAcT*).

**How to cite this article**: Ding, B.-J. *et al*. A plant factory for moth pheromone production. *Nat. Commun.* 5:3353 doi: 10.1038/ncomms4353 (2014).

## Figures and Tables

**Table 1:** Characterization of candidate genes for insect pheromone production in plants.

**Genes**	**Substrate**	**Product**	Conversion rate (%)*
*CpFATB2*	14:ACP	14:acyl	High†
*Atr*Δ*11*	16:CoA	Z11-16:acyl	49.5±2.8‡
*Ave*Δ*11*	14:CoA	E11-14:acyl	9.3±0.5‡
		Z11-14:acyl	33.0±1.9‡
*Cro*Δ*11*	14:CoA	E11-14:acyl	16.5±0.2‡
		Z11-14:acyl	36.9±0.7‡
*CpaE11*	14:CoA	E11-14:acyl	22.7±0.8‡
*Onu*Δ*11*	14:CoA	E11-14:acyl	ND§
		Z11-14:acyl	ND§
*HarFAR*	14:CoA	14:OH	4.6±1.0||
	E11-14:CoA	E11-14:OH	11.3±2.3¶
	Z11-14:CoA	Z11-14:OH	7.8±1.7#
	16:CoA	16:OH	0.4±0.1**
	Z11-16:CoA	Z11-16:OH	1.1±0.3††
*HarFAR_KKYR*	14:CoA	14:OH	14.5±2.8||
	E11-14:CoA	E11-14:OH	28.2±5.8¶
	Z11-14:CoA	Z11-14:OH	19.5±3.9#
	16:CoA	16:OH	1.0±0.1**
	Z11-16:CoA	Z11-16:OH	5.2±1.3††
*YroFAR*	14:CoA	14:OH	0.9±0.5‡
	E11-14:CoA	E11-14:OH	2.1±1.6‡
	Z11-14:CoA	Z11-14:OH	0.4±0.2‡
	16:CoA	16:OH	0.3±0.1‡
	Z11-16:CoA	Z11-16:OH	0.1±0.0‡
*OnuFAR_Z*	Z11-14:CoA	Z11-14:OH	<0.1±0.0‡
*OnuFAR_E*	E11-14:CoA	E11-14:OH	ND§
*EaDAcT*	14:OH	14:OAc	7.0±2.7‡
	E11-14:OH	E11-14:OAc	17.9±5.5‡
	Z11-14:OH	Z11-14:OAc	13.0±1.6‡
	16:OH	16:OAc	6.0±1.4‡
	Z11-16:OH	Z11-16:OAc	7.6±1.7‡

The attachment of KKYR retention signal after the HarFAR resulted in better conversion rate when compared with the wild-type one, tested by *t*-test.

^*^conversion rate=(product/(product+remaining substrate))*100%±s.e.

^†^Amount of 14:acyl (produced from naturally occurring 14:ACP) was *ca*. 100-fold higher in treated compared with control plants.

^‡^±s.e. *n*=3.

^§^ND, not detectable.

^||^*P*=0.029, *n*=4, two sample *t*-tests.

^¶^*P*=0.042, *n*=4, two sample *t*-tests.

^#^*P*=0.039, *n*=4, two sample *t*-tests.

^**^P=0.005, *n*=4, two sample *t*-tests.

^††^*P*=0.037, *n*=4, two sample *t*-tests.

**Table 2:** Production of plant-derived pheromones.

	**14:OAc**	**E11-14:OAc**	**Z11-14:OAc**	**16:OAc**	**Z11-16:OAc**
*Amount of OAc*					
Plant 72 (μg)	301	72	161	64	
Plant 76 (μg)				115	1,280
					
Ratio from plant 72 for *Y. evonymella*	187	45	100	40	
Ratio OAc of mix plant 72+76 for *Y. padella*	187	45	100	82	400

Amounts of pheromone compounds obtained from acetylating the plant-derived fatty alcohols produced in transiently modified plants 72 and 76 and the calculated relative ratios of acetates (Z11-14:OAc=100) in the treatments used for trapping of *Y. padella* and *Y. evonymella*.
